# Visceral Adipose Tissue as an Independent Predictor of Severe Post‐ERCP Pancreatitis: A Multicenter Retrospective Cohort Study

**DOI:** 10.1002/jhbp.70158

**Published:** 2026-07-24

**Authors:** Shinya Uemura, Takuji Iwashita, Takuya Koizumi, Yosuke Ohashi, Shota Iwata, Akinori Maruta, Keisuke Iwata, Masahito Shimizu

**Affiliations:** ^1^ First Department of Internal Medicine Gifu University Hospital Gifu Japan; ^2^ Department of Gastroenterology Shiga University of Medical Science Otsu Shiga Japan; ^3^ Department of Gastroenterology Gifu Municipal Hospital Gifu Japan

**Keywords:** body composition, ERCP, pancreatitis severity, post‐ERCP pancreatitis, visceral adipose tissue

## Abstract

**Background:**

Acute pancreatitis after endoscopic retrograde cholangiopancreatography (ERCP), known as post‐ERCP pancreatitis (PEP), is a major adverse event. Although risk factors for PEP incidence have been widely studied, factors associated with severe PEP remain unclear.

**Aim:**

To identify risk factors, including body composition, associated with severe PEP.

**Methods:**

A retrospective cohort study was conducted in patients who underwent ERCP at two tertiary care centers in Japan between January 2013 and October 2021. PEP severity was defined according to the American Society of Gastrointestinal Endoscopy Workshop criteria (2010). Patients were divided into mild and moderate‐to‐severe groups. Body composition parameters, including skeletal muscle index, subcutaneous adipose tissue, and visceral adipose tissue (VAT), were assessed using CT. Multivariate analysis was performed to identify factors associated with severe PEP.

**Results:**

Among 3087 patients who underwent ERCP for biliary disease, 85 (2.75%) developed PEP. VAT was significantly higher in the severe than mild group (132.7 vs. 80.1 cm^2^, *p* < 0.01). VAT > 95 cm^2^ was the only independent risk factor for severe PEP (OR 2.79, *p* = 0.04).

**Conclusion:**

VAT may be associated with severe PEP. ERCP should be performed with awareness of the increased risk of severe PEP in patients with high visceral adiposity.

## Highlight

Uemura and colleagues found that, among patients with post‐ERCP pancreatitis, visceral adipose tissue area was significantly greater in the moderate‐to‐severe group than in the mild group. An area >95 cm² was the only independent predictor of moderate‐to‐severe pancreatitis, suggesting that pre‐procedural assessment of visceral adiposity may facilitate risk stratification and individualized management.

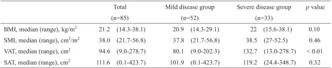

**Abbreviations**
BDS: bile duct stones
BMI: Body mass index
CI: confidence interval
CT: computed tomography
DBE: double‐balloon enteroscopy
DGW: double guidewire
ERCP: Endoscopic retrograde cholangiopancreatography
EUS: endoscopic ultrasound
HU: hounsfield unit
L3: the level of the third lumbar vertebra
MBO: malignant biliary obstruction
NSAIDs: nonsteroidal anti‐inflammatory drugs
OR: odds ratio
PEP: post‐ERCP pancreatitis
SAT: subcutaneous adipose tissue
SMA: skeletal muscle area
SMI: skeletal muscle index
VAT: visceral adipose tissue
WC: waist circumference

## 1 Introduction

Endoscopic retrograde cholangiopancreatography (ERCP) is a widely used minimally invasive procedure to manage biliary diseases, particularly bile duct stones (BDS) and malignant biliary obstruction (MBO). However, ERCP carries the risk of adverse events (AEs), including post‐ERCP pancreatitis (PEP), biliary infection, and perforation. PEP remains a significant concern, with an incidence of 3.5%–10%. Severe cases account for 0.4%–0.5% of cases, with a fatality rate of 0.02%–0.7% [1, 2]. Several studies have identified patient‐ and procedure‐related risk factors for PEP. However, studies specifically investigating the risk factors for severe PEP are limited. Therefore, the present study focused on identifying factors associated with the progression to clinically significant PEP severity rather than the incidence of PEP itself.

Several studies have evaluated the association between body composition and the incidence or severity of acute pancreatitis [3]. Adipose tissue, particularly visceral adipose tissue (VAT) surrounding the abdominal organs, appears to play a crucial role. Yashima et al. demonstrated that excess VAT increases the severity of acute pancreatitis [3]. Although a few studies have explored the relationship between adipose tissue and incidence of PEP, none have specifically focused on the development of severe PEP [4]. Early diagnosis and severity assessment of PEP are important, because even mild cases can progress to severe and potentially fatal pancreatitis. Therefore, identifying the risk factors of severe PEP before ERCP is essential to facilitate early intervention and optimize patient outcomes.

We conducted this study to investigate the risk factors, including body composition analysis for the development of severe PEP. We hypothesized that specific body composition parameters, particularly VAT, might be associated with an increased risk of severe PEP after ERCP.

## 2 Methods

### 2.1 Patients' Selection

This retrospective cohort study was conducted at two tertiary care centers in Japan (Gifu University Hospital and Gifu Municipal Hospital). A database of patients who underwent ERCP between January 2013 and October 2021 was analyzed to identify patients who met the following inclusion and exclusion criteria: Inclusion criteria were patients who underwent ERCP for biliary diseases and subsequently developed PEP. Exclusion criteria were: (1) patients without abdominal computed tomography (CT) imaging within 1 month before ERCP; (2) patients who underwent endoscopic ultrasound (EUS)‐guided biliary drainage (e.g., EUS‐hepaticogastrostomy, EUS‐choledochoduodenostomy, EUS‐rendezvous, or EUS‐antegrade techniques). This study was conducted in accordance with the ethical principles of the Declaration of Helsinki. The study protocol was approved by the Institutional Review Board of each participating hospital (approval number: 2025‐360).

### 2.2 ERCP Procedures

ERCP was performed using a standard duodenoscope (TJF‐290 V or 260 V; Olympus, Tokyo, Japan). A cannula‐preloaded guidewire was gently inserted into the papilla, aligned with the bile duct axis, and carefully advanced into the bile duct, with or without contrast injection. Salvage cannulation techniques, such as double‐guidewire (DGW) or pre‐cutting techniques, are employed when standard cannulation is challenging [5, 6]. Prophylactic pancreatic duct stent placement was performed at the discretion of the endoscopist. Double‐balloon enteroscopy (DBE) (EN‐450 T5; Fujifilm, Tokyo, Japan) was used for patients with altered gastrointestinal anatomy (e.g., Billroth II gastrojejunostomy or Roux‐en‐Y reconstruction). Following biliary cannulation, appropriate papillary and biliary interventions were performed based on underlying biliary disease. Rectal administration of nonsteroidal anti‐inflammatory drugs (NSAIDs) for the prevention of PEP was performed at the discretion of the attending physician. All patients were hospitalized for at least 2 days after the procedure. Serum amylase levels were routinely measured before the procedure, 3 h after the procedure, and on the following day. Procedural data (cannulation methods, success rate, number of attempts, cannulation time, pancreatic duct injections, guidewire insertions, interventions, and total procedure time) and outcome data (clinical symptoms, serum amylase levels, adverse events, and severity) were collected from medical records.

### 2.3 Body Composition Analysis

The body mass index (BMI; kg/m^2^) was calculated as weight (kg) divided by height squared. Visceral adipose tissue (VAT; cm^2^), subcutaneous adipose tissue (SAT; cm^2^), and skeletal muscle area (SMA; cm^2^) were measured from abdominal CT images obtained within 1 month of ERCP. The SMA was assessed using a single axial image at the level of the third lumbar vertebra (L3) [7]. The VAT and SAT were determined from a single axial image at the umbilical level [8]. Body composition analysis was performed using SYNAPSE VINCENT software (Fujifilm), which provides semi‐automated quantification based on predefined Hounsfield unit (HU) thresholds. Slice selection was performed manually, and minor manual adjustments were applied when necessary. Skeletal muscle was defined as −29 to +150 HU, and adipose tissue as −190 to −30 HU. The SMA, VAT, and SAT were automatically calculated by summing the tissue pixels within the defined HU thresholds and multiplying by the pixel surface area [9] (Figure 1). All measurements were performed by a single experienced operator. The skeletal muscle index (SMI; cm^2^/m^2^) was calculated by normalizing the SMA to height.

**FIGURE 1:**
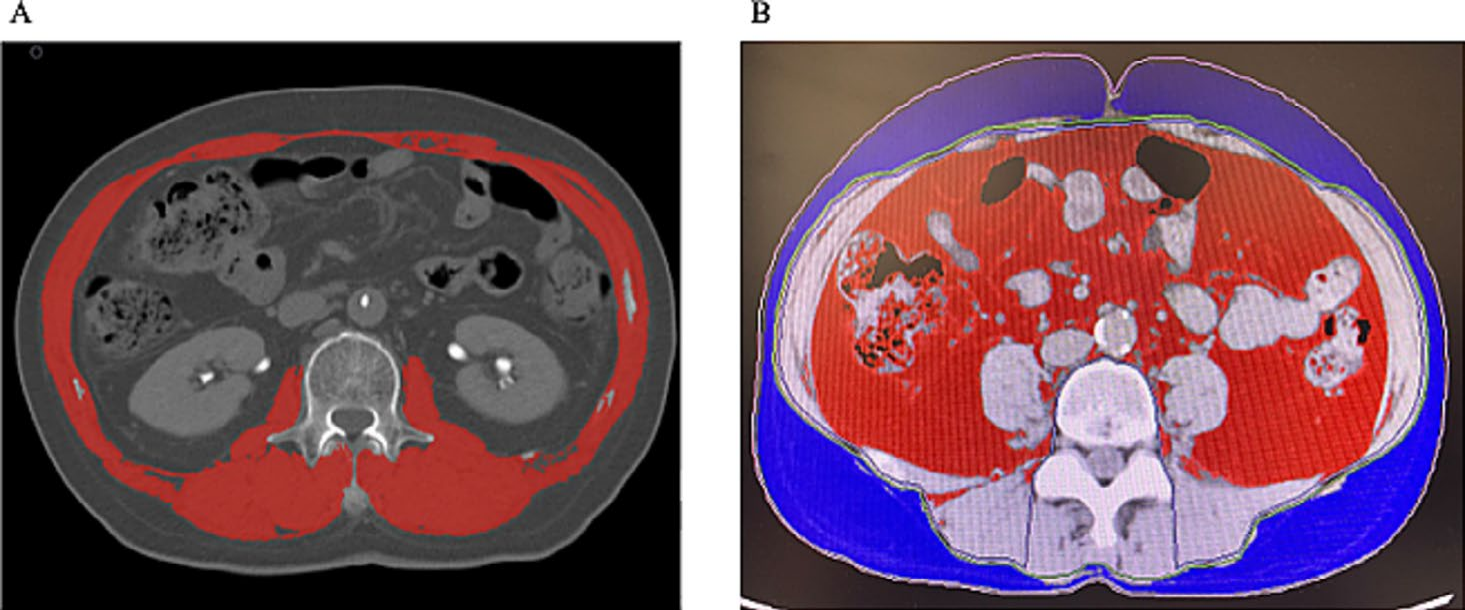
Representative CT images used for body composition analysis. (A) Skeletal muscle area meaured at the level of the third lumber vertebra (L3), with skeletal muscle shown in red. (B) Visceral and subcutaneous adipose tissue measured at the umbilical level, with visceral adipose tissue shown in red and subcutaneous adipose tissue in blue. The color coding differs between panels. The images shown are representative of body composition measurements.

### 2.4 Definitions

Several variables were defined as follows: common bile duct diameter, defined as the maximum diameter measured at the widest portion of the extrahepatic bile duct using pre‐procedural CT imaging; intact papilla, defined as the absence of prior endoscopic sphincterotomy; trainee, defined as an endoscopist with limited experience in ERCP procedures who performed ERCP under the supervision of an experienced endoscopist; time to biliary cannulation, defined as the time from cannulation initiation to successful selective catheterization of the bile duct; total procedure time, defined as the duration from scope insertion to scope removal; ERCP‐related adverse events, defined according to the criteria established by the American Society of Gastrointestinal Endoscopy workshop in 2010; PEP, defined as abdominal pain persisting for ≥ 24 h after ERCP with serum amylase levels > 3 times the upper limit of normal at 18–24 h post‐procedure; PEP severity, classified as mild (< 3 days hospitalization), moderate (4–10 days hospitalization), or severe (> 10 days hospitalization, intensive care unit admission, or surgical intervention), where hospitalization duration was defined as the length of hospital stay from ERCP to discharge; and asymptomatic hyperamylasemia, defined as amylase levels > 3 times the upper limit of normal at 18–24 h post‐ERCP without clinical evidence of PEP [10, 11]. Patients with prior endoscopic sphincterotomy and those undergoing repeated ERCP procedures, including stent exchange, were not excluded from the analysis. Patients were divided into a mild group (mild PEP) and a severe group (moderate and severe PEP combined).

### 2.5 Study Outcomes and Statistical Analysis

The primary outcome was the factors associated with progression to severe PEP. The secondary outcome was the relationship between PEP severity and body composition. The baseline characteristics are presented as medians and ranges. Group comparisons were performed using Fisher's exact test for categorical variables and the Mann–Whitney *U* test for continuous variables. Univariate and multivariate logistic regression analyses were conducted to identify risk factors for severe PEP. Odds ratios (OR) and 95% confidence intervals (CI) were also determined. Factors with *p* < 0.10 in univariate analysis, and established PEP risk factors were included in the multivariate analysis. Statistical significance was set at *p* < 0.05. Continuous variables were dichotomized based on their median values to facilitate analysis in this relatively small cohort. Analyses were performed using JMP software (version 18; SAS Institute, Cary, NC, USA).

## 3 Results

Database analysis identified 3821 patients who underwent ERCP during the study period. After applying the inclusion and exclusion criteria (ERCP for pancreatic disease, EUS‐assisted ERCP, and absence of preoperative abdominal CT), 85 patients who developed PEP after ERCP for biliary disease were included in the analysis (Figure 2). The overall incidence of PEP among the 3087 patients who underwent ERCP for biliary disease was 2.75% (85/3087). Of the 85 patients who developed PEP, 54 (63.5%) were from Gifu University Hospital and 31 (36.5%) were from Gifu Municipal Hospital. The severity of PEP was classified as mild in 52 patients (52/3087, 1.68%), moderate in 26 (26/3087, 0.84%), and severe in 7 (7/3087, 0.23%) (Table 1).

**FIGURE 2:**
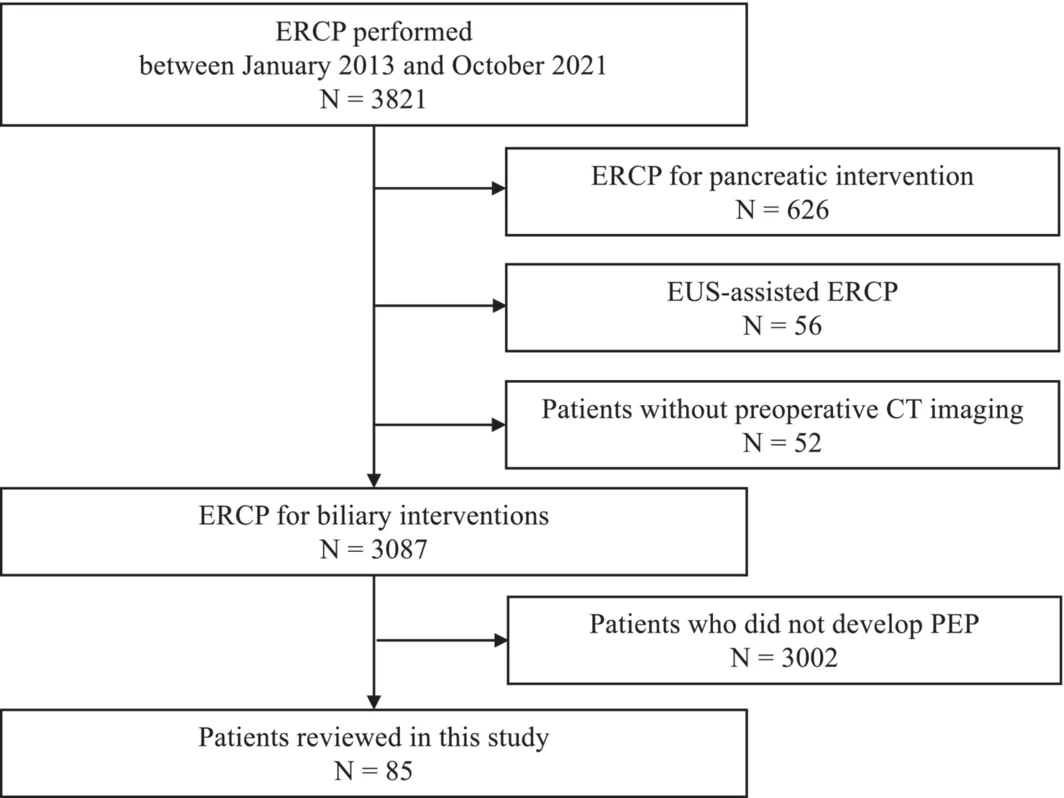
Flowchart of patients included in the study.

**TABLE 1:** Incidence of post‐ERCP pancreatitis.

Patients undergoing ERCP	No.	(%)
Total	3087	
Pancreatitis incidences	85	(2.75%)
Mild pancreatitis incidences	52	(1.68%)
Moderate pancreatitis incidences	26	(0.84%)
Severe pancreatitis incidences	7	(0.23%)

### 3.1 Baseline Characteristics

The characteristics of the 85 patients who developed PEP are summarized in Table 2. The median values for BMI, SMI, VAT, and SAT were 21.2 kg/m^2^ (14.3–38.1), 38.0 cm^2^/m^2^ (21.7–56.8), 94.6 cm^2^ (9.0–278.7), and 111.6 cm^2^ (0.1–423.7), respectively. The indications for ERCP were choledocholithiasis (*n* = 32), biliary malignancy, including pancreatic cancer‐related obstructive jaundice (*n* = 43), and benign biliary disorders (*n* = 10). The details of the ERCP procedures are listed in Table 3. Procedure time was significantly longer in the severe PEP group (*p* = 0.02).

**TABLE 2:** Characteristics of patients with post‐ERCP pancreatitis.

	Total (*n* = 85)	Mild disease group (*n* = 52)	Severe disease group (*n* = 33)	*p*
Age, median (range), years	77 (29–88)	77 (36–89)	77 (29–88)	0.69
Female gender, no. (%)	41 (48.2)	25 (48.1)	16 (48.5)	1.00
Reasons for ERCP, no. (%)				
Choledocholithiasis	32 (37.6)	17 (32.7)	15 (45.5)	0.26
Biliary malignancy	43 (50.6)	29 (55.8)	14 (42.4)	0.27
Benign biliary disorder	10 (11.8)	6 (11.5)	4 (12.1)	1.00
Pancreatic cancer, no. (%)	12 (14.1)	9 (17.3)	3 (9.1)	0.35
Billroth II or R‐Y, no. (%)	4 (4.7)	2 (3.9)	2 (6.1)	0.64
Intact papilla, no. (%)	77 (90.6)	46 (88.5)	31 (93.9)	0.46
Periampullary diverticulum, no. (%)	18 (21.2)	10 (19.2)	8 (24.2)	0.60
Presence of jaundice at ERCP, no. (%)	43 (50.6)	28 (53.9)	15 (45.5)	0.51
CBD diameter, median (range), mm	10 (6–25)	10 (6–25)	10 (6–20)	0.97

Abbreviations: Billroth II, Billroth II gastrojejunostomy; BMI, body mass index; CBD, common bile duct; R‐Y, Roux‐en‐Y reconstruction; SAT, subcutaneous adipose tissue; SMI, skeletal mass index; VAT, visceral adipose tissue.

**TABLE 3:** Procedures performed during ERCP for patients with post‐ERCP pancreatitis.

	Total (*n* = 85)	Mild disease group (*n* = 52)	Severe disease group (*n* = 33)	*p*
Papillary manipulation, no (%)				
Sphincterotomy	55 (64.7)	33 (63.5)	22 (66.7)	0.82
Balloon dilation	3 (3.5)	2 (3.9)	1 (3.0)	1.00
Large balloon dilation	9 (10.6)	4 (7.7)	5 (15.2)	0.30
None	18 (21.2)	13 (25.0)	5 (15.2)	0.42
Cannulation method, no. (%)				
Contrast or Wire‐guided cannulation	48 (56.5)	33 (63.5)	15 (45.5)	0.12
Double guidewire cannulation technique	28 (32.9)	15 (28.9)	13 (39.4)	0.35
Pre‐cutting	9 (10.6)	4 (7.7)	5 (15.2)	0.30
Attempt time, median range, min	10 (2–60)	8 (2–42)	12 (2–60)	0.10
Procedure time, median range, min	28 (6–100)	24 (8–73)	36 (6–100)	0.02
ERCP by trainees, no. (%)	47 (55.3)	27 (51.9)	20 (60.6)	0.51
Unintentional PD manipulation, no. (%)				
Contrast material injection	50 (58.8)	28 (53.9)	22 (66.7)	0.27
Guidewire insertion	38 (44.7)	19 (36.5)	19 (57.6)	0.08
Prophylactic PD stent, no. (%)	3 (3.5)	2 (3.9)	1 (3.0)	1.00
Nasobiliary drainage, no. (%)	28 (32.9)	19 (36.5)	9 (27.3)	0.48
Plastic stent, no. (%)	9 (10.6)	7 (13.5)	2 (6.1)	0.47
Uncovered metal stent, no. (%)	1 (1.2)	0	1 (3.0)	0.39
Covered metal stent, no. (%)	20 (23.5)	12 (23.1)	8 (24.2)	1.00
Transpapillary biliary stricture biopsy, no. (%)	16 (18.8)	11 (21.2)	5 (15.2)	0.58
Peroral cholangioscopy, no. (%)	5 (5.9)	1 (1.92)	4 (12.1)	0.07
Rectal NSAIDs administration	4 (5.0)	3 (5.8)	1 (3.0)	1.00

Abbreviations: NSAIDs, nonsteroidal anti‐inflammatory drugs; PD, pancreatic duct.

### 3.2 Body Composition and Severe PEP

A comparison of body composition parameters between the mild and severe PEP groups is presented in Table 4. Although BMI, SMI, and SAT were similar between the groups, VAT was significantly higher in the severe PEP group (*p* < 0.01). The median VAT values were 80.1 (9.0–202.3) cm^2^ in the mild group and 132.7 (13.0–278.7) cm^2^ in the severe group.

**TABLE 4:** Body composition values in mild and severe disease groups of PEP.

	Total (*n* = 85)	Mild disease group (*n* = 52)	Severe disease group (*n* = 33)	*p*
BMI, median (range), kg/m^2^	21.2 (14.3–38.1)	20.9 (14.3–29.1)	22 (15.6–38.1)	0.10
SMI, median (range), cm^2^/m^2^	38.0 (21.7–56.8)	37.8 (21.7–56.8)	38.5 (27–52.5)	0.46
VAT, median (range), cm^2^	94.6 (9.0–278.7)	80.1 (9.0–202.3)	132.7 (13.0–278.7)	< 0.01
SAT, median (range), cm^2^	111.6 (0.1–423.7)	101.9 (0.1–423.7)	119.2 (24.4–348.7)	0.32

Abbreviations: BMI, body mass index; SAT, subcutaneous adipose tissue; SMI, skeletal mass index; VAT, visceral adipose tissue.

### 3.3 Evaluation of Risk Factors for the Development of Severe PEP

The results of univariate and multivariate analyses of the risk factors for the development of severe PEP are provided in Table 5. The following factors were examined in the univariate analysis: patient characteristics, body composition, and ERCP procedures. Multivariate analysis included 6 variables (4 with *p* < 0.1, univariate analysis: VAT value > 95cm^2^, procedure time > 29 min, guide wire insertion into the pancreatic duct, and use of peroral cholangioscopy and 2 as established risk factors for PEP: young age (< 77 years) and female sex). Multivariate analysis identified VAT > 95 cm^2^ as the only independent predictor of severe PEP (OR: 2.79, 95% CI: 1.03–7.98, *p* = 0.04).

**TABLE 5:** Univariate and multivariate analyses of risk factors for the development of severe PEP.

Variable	Univariate			Multivariate		
	OR	95% CI	*p*	OR	95% CI	*p*
Younger age < 77 years	1.20	0.48–2.93	0.69	1.32	0.49–3.55	0.58
Female gender	1.21	0.50–2.97	0.67	1.12	0.41–3.08	0.82
BMI > 22 kg/m^2^	1.68	0.69–4.13	0.26			
SMI < 38 cm^2^/m^2^	0.88	0.36–2.19	0.78			
VAT > 95 cm^2^	2.86	1.16–7.34	0.03	2.79	1.03–7.98	0.04
SAT > 110 cm^2^	1.33	0.55–3.26	0.52			
Reason for ERCP						
Choledocholithiasis	Ref.					
Biliary malignancy	0.58	0.23–1.40	0.23			
Benign biliary disorder	0.72	0.15–2.82	0.65			
Pancreatic cancer	0.30	0.04–1.26	0.11			
Jaundice at ERCP	0.58	0.23–1.40	0.23			
Intact papilla	2.02	0.43–14.4	0.39			
Periampullary diverticulum	1.14	0.38–3.29	0.81			
ERCP by trainees	1.47	0.60–3.67	0.39			
Billroth II or R‐Y	1.79	0.21–15.58	0.57			
Common bile duct diameter ≤ 10 mm	0.64	0.25–1.57	0.33			
Procedure time > 29 min	2.30	0.94–5.81	0.07	1.61	0.57–4.60	0.37
Attempt time > 10 min	1.61	0.66–3.98	0.30			
Cannulation method						
Contrast or Wire‐guided cannulation	Ref.					
Double guidewire cannulation technique	1.88	0.74–4.79	0.18			
Pre‐cutting	1.45	0.34–5.94	0.60			
Papillary manipulation						
Sphincterotomy	Ref.					
Balloon dilation	0.87	0.04–9.41	0.91			
Large balloon dilation	2.40	0.59–10.45	0.22			
None	0.61	0.18–1.81	0.38			
PD contrast material injection	1.45	0.59–3.69	0.42			
PD guidewire insertion	2.35	0.96–5.91	0.06	1.51	0.53–4.32	0.44
Nasobiliary drainage	0.59	0.21–1.58	0.28			
Plastic stent	0.46	0.07–2.07	0.33			
Covered metal stent	0.92	0.31–2.58	0.88			
Transpapillary biliary stricture biopsy	0.75	0.22–2.32	0.63			
Peroral cholangioscopy	7.85	1.10–157.59	0.04	7.85	0.82–184.25	0.08
Rectal NSAIDs administration	0.51	0.02–4.18	0.55			

*Note:* Intact papilla was coded as 1 and non‐intact papilla as 0.

Abbreviations: NSAIDs, nonsteroidal anti‐inflammatory drugs; PD, pancreatic duct.

## 4 Discussion

Our study evaluated the potential factors, including body composition, associated with the development of severe PEP. Of the 3821 patients who underwent ERCP, 85 (2.75%) developed PEP. The severity of PEP was classified as mild in 52 patients (52/3087:1.68%), moderate in 26 (26/3087:0.84%), and severe in 7 (7/3087:0.23%). Multivariate analysis showed that only VAT was an independent risk factor for moderate or severe PEP (OR: 2.79, 95% CI: 1.03–7.98, *p* = 0.04).

Although numerous studies have identified risk factors for the incidence of PEP, which are primarily categorized as patient‐ and procedure‐related, the risk factors for PEP severity remain poorly defined and may differ from those associated with incidence. The procedure‐related risk factors for PEP include challenging biliary cannulation, pre‐cut sphincterotomy, pancreatic sphincterotomy, pancreatic duct injection, and retained bile duct stones [12, 13, 14, 15, 16]. The patient‐related risk factors include younger age, female sex, history of recurrent pancreatitis, suspected sphincter Oddi dysfunction, absence of chronic pancreatitis, absence of bile duct dilation, and obesity (higher BMI) [1, 16, 17]. Our study found no association between these factors and PEP severity. This suggests that the factors contributing to PEP development may not be the primary determinants of subsequent severity.

As risk factors for the severity of PEP, the current study showed that higher VAT was associated with the severity of PEP, although other parameters, such as body weight, BMI, and SAT, were not significant factors. This finding aligns with previous research demonstrating a link between obesity and the severity of pancreatitis. According to a meta‐analysis by Martinez et al., which investigated whether obesity can be a risk factor for acute pancreatitis and included 739 cases, obesity (BMI > 30 kg/m^2^) was a significant risk factor for severity (OR: 2.9, 95% CI: 1.8–4.6), development of local complications (OR: 3.8, 95% CI: 2.4–6.6), systemic complications (OR: 2.3, 95% CI: 1.4–4.8), and mortality (OR: 2.1, 95% CI: 1.0–4.8) [18]. The fat distribution varies among obese patients. BMI, VAT, SAT, and waist circumference (WC) are some of the most common indicators of obesity. Yashima et al. conducted a retrospective study with acute pancreatitis, investigated the relationship between various obesity assessment indicators (BMI, VAT, SAT, and WC) and the severity of acute pancreatitis in 124 patients. They reported that BMI, VAT, SAT, and WC were correlated with severity of acute pancreatitis in univariate analysis. However, only VAT strongly correlated with severe acute pancreatitis in the multivariate analysis (OR: 1.02, 95% CI: 1.01–1.03, *p* = 0.003). These studies, including ours, indicate that the distribution of adipose tissue, specifically the amount of visceral fat, could be more important in estimating the severity of PEP than just focusing on body weight or BMI.

Several mechanisms may explain the association between VAT and the severity of pancreatitis. In acute pancreatitis, activated pancreatic enzymes can cause autodigestion of the pancreas and surrounding adipose tissue, leading to visceral fat necrosis. This necrosis can trigger pseudocyst formation and release of inflammatory cytokines, potentially contributing to a more severe clinical course. Necrotic visceral fat can become encapsulated, forming pseudocysts that may become infected and complicate the management of pancreatitis [19, 20, 21]. In addition, extensive visceral fat necrosis can release large amounts of inflammatory cytokines, potentially leading to systemic inflammatory response syndrome and multi‐organ dysfunction. Several studies have reported that the serum concentrations of inflammatory cytokines are elevated in patients with acute pancreatitis [22, 23, 24]. Therefore, excessive visceral adiposity may amplify inflammatory cascades once pancreatitis is triggered, leading to a more severe clinical course. According to a report by Yashima et al., a significant correlation between VAT volume and APACHE II score, a widely used scoring system to assess disease severity in adult patients admitted to intensive care units, was noted in patients with acute pancreatitis [3]. Our findings suggested that a similar mechanism may be involved in PEP, with higher VAT levels increasing the risk of a more severe and complicated clinical course.

Various methods have been considered to prevent the incidence of PEP. A meta‐analysis comparing rectal NSAIDs with a placebo showed a significant reduction in the incidence of PEP with rectal NSAIDs (OR: 0.46, 95% CI: 0.32–0.70). The incidences of moderate and severe pancreatitis, as classified using the Cotton severity scale, were also significantly reduced (OR: 0.46, 95% CI: 0.27–0.77) [25]. This suggests that the administration of NSAIDs may help prevent the progression of PEP to more severe forms. In this study, the rectal administration of NSAIDs was not routinely performed, and only a minimal number of patients received this treatment at the discretion of the attending physician. Furthermore, some reports indicated that temporary prophylactic pancreatic duct stenting is effective in preventing the onset of PEP. The meta‐analysis showed that the incidence of PEP was significantly reduced by temporary placement of a pancreatic duct stent (OR: 0.32; 95% CI: 0.23–0.45). The incidence of severe pancreatitis was also significantly reduced (OR: 0.21, 95% CI: 0.10–0.45), which suggests that temporary pancreatic duct stenting may contribute to preventing PEP severity [25]. This study was performed at the discretion of the attending physician to determine whether a prophylactic pancreatic duct stent should be placed. Prophylactic temporary pancreatic duct stenting may be recommended for patients who are at high risk of developing PEP or who are at high risk of severe PEP. Although other reports have suggested the usefulness of rapid aggressive hydration before and after ERCP, due to a lack of clear evidence, further investigations are needed [25, 26].

Given the association between VAT and PEP severity, we recommend the preoperative assessment of body fat content, particularly VAT, using CT or other imaging modalities to identify patients at higher risk before performing ERCP. In patients with a high‐volume VAT, temporary pancreatic duct stenting should be considered when performing high‐risk procedures for PEP development. Furthermore, proactive measures to prevent PEP, such as the administration of rectal NSAIDs and aggressive hydration, should be considered. If PEP occurs, meticulous management with heightened awareness of the potential development of severe PEP is crucial.

The limited number of patients with severe PEP reflects the inherently low incidence of this complication in routine clinical practice. Accordingly, moderate and severe PEP were combined to define clinically significant PEP, an approach consistent with previous studies focusing on disease severity [10]. However, these categories represent clinically distinct outcomes, and therefore, the interpretation of “severe” PEP in this study should be made with caution. The present study has several limitations. First, this was a retrospective study with a relatively small cohort size from only two centers, which may have introduced bias in patient selection or treatment strategy. Second, data regarding specific complications such as walled‐off necrosis were not available. Third, although we attempted to reduce potential bias by enrolling consecutive patients and conducting multivariate analysis, prophylactic measures, including rectal NSAIDs administration and pancreatic duct stenting, were not standardized and were left to the discretion of the endoscopist. As these interventions may influence the severity of PEP, residual confounding cannot be excluded. In addition, body composition analysis using semi‐automated software may be subject to operator dependency, particularly in slice selection and manual adjustment, and interobserver variability was not assessed in this study. Furthermore, the VAT cutoff value was based on the median of the study population, allowing pragmatic dichotomization in this relatively small cohort. This approach may have reduced statistical power and introduced bias. Moreover, this cutoff was not based on established clinical criteria and may limit external validity. Future prospective studies are warranted to establish clinically meaningful threshold values. Visual differences in VAT were not specifically illustrated, as the analysis was based on quantitative assessment. Our findings should be interpreted within the context of patients who developed PEP. The identified factors are associated with progression to severe PEP among patients with PEP, and do not directly represent predictors of severe PEP in the overall ERCP population.

In conclusion, VAT may be associated with progression to severe PEP among patients who develop PEP. In patients with high VAT, careful monitoring and appropriate management may help prevent progression to severe disease. Pre‐procedural assessment of visceral adiposity may facilitate risk stratification and inform individualized management strategies. However, the effectiveness of specific prophylactic interventions was not evaluated, and these findings should be considered hypothesis‐generating.

## Author Contributions

S.U.: Conceptualization, data curation, methodology, validation, formal analysis, investigation, writing – original draft, visualization. T.I.: Conceptualization, methodology, writing – review and editing, project administration. T.K., Y.O., S.I., A.M., and K.I.: Investigation, visualization. M.S.: Supervision. All authors read and approved the final manuscript.

## Conflicts of Interest

The authors declare no conflicts of interest.

## Data Availability

The data that support the findings of this study are available from the corresponding author upon reasonable request.
